# Mitochondrial sirtuins: Energy dynamics and cancer metabolism

**DOI:** 10.1016/j.mocell.2024.100029

**Published:** 2024-02-06

**Authors:** Hojun Lee, Haejin Yoon

**Affiliations:** Department of Biological Sciences, Ulsan National Institute of Science and Technology, Ulsan 44919, Republic of Korea

**Keywords:** Cancer metabolism, Mitochondrial sirtuins, Mitochondria metabolism, NAD+, SIRT3

## Abstract

Mitochondria are pivotal for energy regulation and are linked to cancer. Mitochondrial sirtuins, (Sirtuin) SIRT3, SIRT4, and SIRT5, play crucial roles in cancer metabolism. This review explores their impact on cellular processes, with a focus on the NAD+ interplay and the modulation of their enzymatic activities. The varied roles of SIRT3, SIRT4, and SIRT5 in metabolic adaptation and cancer are outlined, emphasizing their tumor suppressor or oncogenic nature. We propose new insights into sirtuin biology, and cancer therapeutics, suggesting an integrated proteomics and metabolomics approach for a comprehensive understanding of mitochondrial sirtuins in cancer.

## INTRODUCTION

Mitochondria play crucial roles in energy production, metabolism, and cell signaling (Mitchell, 1961). Dysregulation of these organelles is linked to diseases like cancer, neurodegenerative disorders, and metabolic anomalies (Aparicio et al., 2022, Reid et al., 2022). Specifically, mitochondria regulate cellular energy through adenosine triphosphate (ATP) and related metabolites, including nicotinamide adenine dinucleotide (NAD+). Sirtuins, NAD+-dependent protein deacetylases, have garnered attention for their involvement in aging, metabolism, and longevity (Mouchiroud et al., 2013). The mammalian sirtuin family, comprising 7 isoforms localized in different cellular compartments, plays a crucial role in gene expression, DNA repair, cellular senescence, and mitochondrial function (Bosch-Presegué and Vaquero, 2014; Hamaidi and Kim, 2022).

In the cancer context, cells adopt altered metabolic pathways, exemplified by the Warburg effect, providing a growth advantage (Warburg et al., 1927). Mitochondrial sirtuins, especially SIRT3, SIRT4, and SIRT5, emerge as key regulators of cancer metabolism. SIRT3, the major mitochondrial sirtuin, governs mitochondrial function and cellular bioenergetics, impacting fatty acid oxidation, amino acid metabolism, and the tricarboxylic acid cycle (Bharathi et al., 2013, Scher et al., 2007). It promotes oxidative phosphorylation, contributing to the inhibition of tumor cell proliferation and metastasis (Finley et al., 2011, Lee et al., 2018). SIRT4 inhibits crucial glutamine metabolism for cancer cell growth, affecting metabolite availability and lipid metabolism (Haigis et al., 2006). SIRT5 modulates metabolic pathways through protein modification, influencing cancer cell metabolism and survival (Nakagawa et al., 2009). Cofactor NAD+ plays a critical role in cellular redox reactions and energy metabolism, serving as a substrate for sirtuin enzymatic activity (Greiss and Gartner, 2009). The interplay between mitochondrial sirtuins and NAD+ significantly influences cellular metabolism and cancer cell survival.

While mitochondrial sirtuins share conserved sequence-based domains, variations exist in their enzymatic activity and substrates (Frye, 2000). To unravel this intricate interplay and comprehend their distinct roles in metabolic diseases, including cancer, comprehensive approaches involving their interactome and NAD+ metabolism are imperative. Here, we provide an overview of the current literature on mitochondrial sirtuins and their evolving role in cancer metabolism. It explores the specific functions of SIRT3, SIRT4, and SIRT5 in regulating cellular metabolism and their impact on NAD+ consumption. Additionally, it investigates the implications of mitochondrial sirtuins for cancer cell survival and proliferation, offering insights critical for the development of targeted therapies in mitochondrial sirtuin or NAD+ biology for cancer treatment.

## EXPLORING CELLULAR ENERGY SENSING THROUGH THE INTRICACIES OF ATP AND NAD+ INTERPLAY

ATP serves as the vital energy carrier and plays a central role in essential cellular processes, including DNA and RNA synthesis (Beis and Newsholme, 1975). ATP is generated through glycolysis and oxidative phosphorylation, with the electron transport chain fueled by NADH and flavin adenine dinucleotide (FADH2), and its synthesis rates are finely tuned by adenosine monophosphate (AMP)-activated protein kinase in response to the cell's metabolic needs (MITCHELL, 1961, Oakhill et al., 2011).

NADH, NADPH, and ATP are essential for gauging cell health. NAD+ plays a leading role in hydrogen transfer, significantly contributing to ATP production through mitochondrial oxidative phosphorylation (OXPHOS) (Warburg et al., 1927). Regulated NAD+ levels involve proteins and enzymes sensitive to dynamic concentrations (Cambronne et al., 2016), with recent studies highlighting the importance of localized NAD+ in mitochondria for controlling cellular functions.

Mitochondrial sirtuins, SIRT3, SIRT4, and SIRT5, emerge as NAD+-dependent protein deacylases exclusively situated in the mitochondrial matrix. Guided by their need for NAD+, these enzymes play a unique role in changing the group of proteins in the mitochondria called lysine acylome (Carrico et al., 2018). We concentrate on the mitochondrial NAD+-dependent enzyme, where NAD+ concentration exceeds that in the nucleus and cytoplasm, rather than investigating how NAD+ attains varying concentrations across cellular organelles. This helps us study how NAD+ affects enzyme activity and learn more about its impact on cellular processes. Additionally, we aim to understand the distinctive roles played by mitochondrial sirtuins in cellular processes, elucidating how their dependence on NAD+ shapes the dynamics of the cell.

## BIOCHEMICAL MECHANISMS OF ACTION OF MITOCHONDRIAL SIRTUINS

### Mode of Action of Mitochondrial Sirtuins Through Molecular Structure

**Fig. 1:**
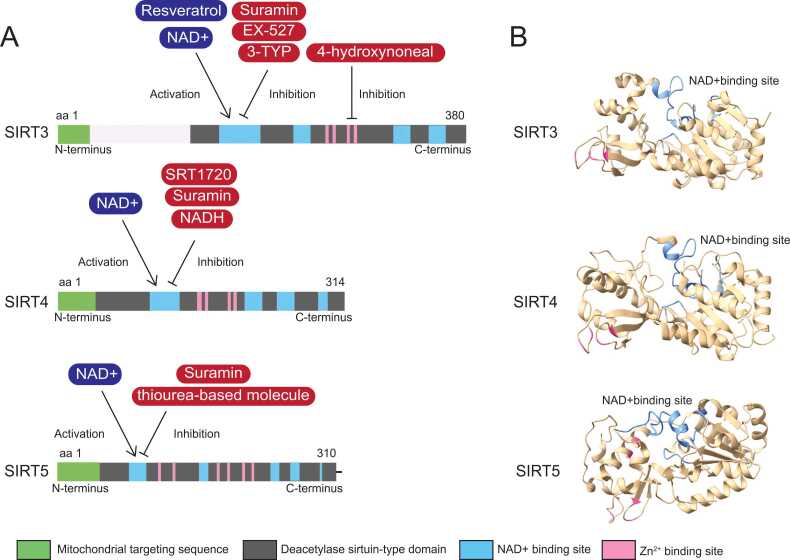
Structural insights and functional domains of mitochondrial sirtuin proteins. (A) Schematic representation of the structural domains of sirtuin proteins. Each sirtuin protein possesses distinct functional domains: Mitochondrial targeting sequence (green), NAD+ binding site (blue), deacetylase sirtuin-type domain (dark gray), and zinc ion binding zone (pink). (B) Molecular structures of key sirtuin proteins—SIRT3 (PDB: 4BV3), SIRT4 (PDB: 5OJN), and SIRT5 (PDB: 6LJK). The structures highlight the intricate arrangement of functional domains crucial for their respective biological activities. aa, amino acid.

Mitochondrial sirtuins, specifically SIRT3, SIRT4, and SIRT5, reveal a shared and structurally conserved deacylase domain featuring key components: catalytic histidine domain, Rossmann-fold domain, zinc-binding domain, and cofactor binding loops region (Ahuja et al., 2007, Anderson et al., 2017, Du et al., 2011, Onyango et al., 2002, Pannek et al., 2017). These proteins also harbor a mitochondrial targeting sequence, guiding them to mitochondria as a 10 to 70 amino acid signal at the N-terminus, later cleaved for activation (Nakagawa et al., 2009) (Fig. 1A).

The catalytic histidine domain plays a vital role by abstracting a proton from the hydroxyl group, enhancing the nucleophilicity of oxygen. The Rossmann-fold domain encompasses an NAD+-binding site with a Gly-X-Gly sequence for phosphate binding, including an inhibitory nicotinamide (NAM) region (Fig. 1A and B). The zinc-binding domain consists of a zinc-binding module and a helical module crucial for structural stability. Cofactor binding loop regions, connecting Rossmann fold and zinc-binding domains, form the cleft serving as the enzyme's active site. Acetyl-lysine and NAD+ are inserted into a hydrophobic tunnel from opposing sides, governed by several invariant amino acids, ensuring substrate binding and catalysis (Zhou et al., 2018).

SIRT3, SIRT4, and SIRT5 share a fundamental chemical reaction, catalyzing NAD+-dependent deacetylation of acetyl-lysine. This reaction yields deacetylated lysine, NAM, and 2′-O-adenosine diphosphate (ADP)-ribose. The process involves cutting the N-glycosidic bond in NAD+ (Hoff and Wolberger, 2005).

Substrate recognition by SIRT3, SIRT4, and SIRT5 primarily involves 4 to 5 target residues N- and C-terminal from acetyl-Lys, interacting through backbone interactions and surface contacts. Notably, SIRT4 exhibits a more subtle deacetylation sequence preference compared to SIRT5 and SIRT3 (Rauh et al., 2013).

### Modulators of Mitochondrial Sirtuins

The NAD+ binding site within sirtuin proteins features distinct subsites facilitating interaction. Interestingly, metabolites from NAD+ biology participate in SIRT3, SIRT4, and SIRT5 enzymatic activity. NAM, the primary NAD+ source in cell lines and murine tissues, inhibits SIRT3 by rebinding to the enzyme, accelerating the reverse reaction (Guan et al., 2014). Beyond metabolites, various Sirtuin-targeted drugs are well studied. Resveratrol is known to increase SIRT3 expression (Xu et al., 2016), as is Metformin (Diaz-Morales et al., 2018). Other activators and inhibitors, including metabolic enzymes and microRNAs, are detailed in Table 1.

**Table 1:** Modulators of mitochondrial sirtuins and their effects

Target	Compound name	Sirtuin effects	Reference
Cellular SIRT3 activator	NAD+	NAD promotes the deacetylation process of SIRT3	Someya et al. (2010)
	Mitochondrial processing peptidase (MPP)	MPP process the full-length SIRT3 protein in the mitochondrial matrix	Schwer et al. (2002)
	Sentrin-specific protease 1 (SENP1)	SENP1 can de-SUMOylates and activates SIRT3	Wang et al. (2019)
	nuclear factor kappa-light-chain-enhancer of activated B cells (NF-kB)	NF-κB binds to the SIRT3 promoter to enhance its expression	Neeli et al. (2020)
	Peroxisome proliferator-activated receptor gamma coactivator 1-alpha (PGC-1*α*)	PGC-1α bounds to the SIRT3 promoter as its transcription factor to regulate SIRT3 expression	Giralt et al. (2011)
	Taurine up-regulated 1 (TUG1)	TUG1 negatively regulates the expression of microRNA (miR)-145 thus indirectly positively regulating SIRT3	Zeng et al. (2017a)
	Long Intergenic Non-Protein Coding RNA 1228 (DYNLRB2-2)	DYNLRB2-2 suppresses the messenger RNA (mRNA) expression of miR-298 thus indirectly activate SIRT3	Li et al. (2019b)
	Profilin-1	Profilin-1 interacts with SIRT3 and promotes its expression	Yao et al. (2014)
	*β*-Catenin	β-catenin suppresses SIRT3 promotor activity to negatively regulate its expression	Jiang et al. (2018)
	Nicotinamide	Nicotinamide inhibits SIRT3 through rebinding of the reaction product to the enzyme accelerates the reverse reaction	Jiang et al. (2017)
		
SIRT3 activator (small molecule)	Honokiol	Honokiol increases SIRT3 expression and activity	Pillai et al. (2015)
	Silybin	Silybin Increases SIRT3 expression	Li et al. (2017)
	Resveratrol	Resveratrol increases SIRT3 expression	Xu et al. (2016)
	Polydatin	Polydatin increases SIRT3 activity	Zhang et al. (2017)
	Dihydromyricetin	Dihydromyricetin increases the expression and activity of SIRT3 via activation of PGC-1α	Wang et al. (2018)
	Pyrroloquinoline	Pyrroloquinoline quinone increases the expression and activity of SIRT3	Zhang et al. (2015)
	Metformin	Metformin increases SIRT3 expression	Diaz-Morales et al. (2018)
	Adjudin	Adjudin increases the expression of SIRT3	Quan et al. (2015)
	Melatonin	Melatonin activates the SIRT3 signaling pathway	Zhai et al. (2017)
	7-Hydroxy-3-(4′-methoxyphenyl) coumarin (C12)	C12 binds to the acetylated manganese superoxide dismutase (MnSODK68AcK)-SIRT3 complex and promotes the deacetylation and activation of MnSOD	Lu et al. (2017)
	Oroxylin A	Oroxylin A could increase the expression or activity of SIRT3	Wei et al. (2013)
	4-Hydroxynonenal	4-Hydroxynonenal inhibits SIRT3 activity by occupying its zinc-binding residue Cys (280)	Fritz et al. (2013)
		
Cellular SIRT3 inhibitor	Snail family transcriptional repressor 1 (SNAI1)	SNAI1 inhibits SIRT3 promoter activity	Zhang et al. (2018a)
	Zinc finger E-box binding homeobox 1 (ZEB1)	ZEB1 inhibits SIRT3 promoter activity	Xu et al. (2018)
	miR-195	miR-195 downregulates SIRT3 expression through direct 3′-untranslated region targeting	Zhang et al. (2018b)
	miR-421	miR-421 targets the 3′ untranslated region (UTR) of SIRT3 and decreases SIRT3 protein level	Cheng et al. (2016)
	miR-494-3p	miR-494-3p targets the 3′UTR of SIRT3 and inhibits SIRT3 expression at mRNA and protein levels	Geng et al. (2018)
	miR-708-5p	miR-708-5p targets the 3′UTR of SIRT3 and decreases SIRT3 protein level	Huang et al. (2019)
	miR-31	miR-31 directly targets SIRT3 to repress its expression	Kao et al. (2019)
	miR-145	miR-31 directly targets SIRT3 to reduce its expression	Zeng et al. (2017a)
	miR-298	miR-298 directly targets SIRT3 to inhibit its expression	Li et al. (2019b)
	miR-210	miR-210 targets and represses iron-sulfur cluster assembly enzyme (ISCU) to change the NAD+/NADH ratio thus indirectly negative regulate SIRT3	Sun et al. (2017)
		
SIRT3 inhibitor (small molecule)	Suramin	Suramin occupy the complete active site of SIRT3	Trapp et al. (2007)
	5-Amino-2-phenyl-benzoxazole	Phenyl moiety of 5-amino-2-phenyl-benzoxazole has significant effects on the inhibitory activity	Salo et al. (2013)
	4′-Bromo-Resveratrol	Competitive inhibitor of SIRT3	Nguyen et al. (2013)
	(4-[(2-Hydroxy-6-phenylnaphthalen-1-yl) methyl]-5-(4-methylphenyl)-2,3-dihydro-1H-pyrazol-3-one)	Competitive inhibitor of SIRT3 in the view of structure activity relationship (SAR)	Mahajan et al. (2014)
	(2S,5S,8S)-5-(4-ethanethioamidobutyl)-2-(naphthalen-2-ylmethyl)-3,6,13,20-tetraoxo-1,4,7,12-tetraazacycloicosane-8-carboxamide	Competitive inhibitor of SIRT3 in the view of structure activity relationship (SAR)	Mahajan et al. (2014)
	(3S,6S,9S)-9-butyl-6-(4-ethanethioamidobutyl)-5,8,11,18-tetraoxo-1,4,7,10-tetraazacyclooctadecane-3-carboxamide	Macrocyclic analogs of Nε-thioacetyl-lysine-containing tripeptide	Cheng et al. (2016)
	(2S,5S,8S)-2-butyl-5-(4-ethanethioamidobutyl)-3,6,12,19-tetraoxo-1,4,7,11-tetraazacyclononadecane-8-carboxamide	Macrocyclic analogs of Nε-thioacetyl-lysine-containing tripeptide	Cheng et al. (2016)
	(2S,5S,8S)-2-butyl-5-(4-ethanethioamidobutyl)-3,6,13,20-tetraoxo-1,4,7,12-tetraazacycloicosane-8-carboxamide	Macrocyclic analogs of Nε-thioacetyl-lysine-containing tripeptide	Cheng et al. (2016)
	(2S,5S,8S)-2-butyl-5-(4-ethanethioamidobutyl)-3,6,14,21-tetraoxo-1,4,7,13-tetraazacyclohenicosane-8-carboxamide	Macrocyclic analogs of Nε-thioacetyl-lysine-containing tripeptide	Chen and Zheng (2016)
	(S)-2-((S)-4-([1,1′-biphenyl]-4-yl)-2-acetamidobutanamido)-N-((S)-6-acetamido-1-amino-1-oxohexan-2-yl)-6-ethanethioamidohexanamide	Analogs of Nε-thioacetyl-lysine	Chen et al. (2015)
	N,N′-((S)-6-(((S)-1-(((S)-4-acetamido-1-amino-1-oxobutan-2-yl)amino)-6-ethanethioamido-1-oxohexan-2-yl)amino)-6-oxohexane-1,5-diyl)diacetamide	Analogs of Nε-thioacetyl-lysine	Chen et al. (2015)
	N,N′-((S)-6-(((S)-1-(((S)-1-amino-1-oxohexan-2-yl)amino)-6-ethanethioamido-1-oxohexan-2-yl)amino)-6-oxohexane-1,5-diyl)diacetamide	Analogs of Nε-thioacetyl-lysine	Chen et al. (2015)
	N,N′-((S)-6-(((S)-1-(((S)-1-amino-4-(naphthalen-2-yl)-1-oxobutan-2-yl)amino)-6-ethanethioamido-1-oxohexan-2-yl)amino)-6-oxohexane-1,5-diyl)diacetamide	Analogs of Nε-thioacetyl-lysine	Chen et al. (2015)
	Small molecule mitochondrial-targeting SIRT3 inhibitor (YC8-02)	Competitive inhibitor of SIRT3	Li et al. (2019a)
	Benzyl (S)-(1-((3-hydroxyphenyl)amino)-1-oxo-6-tetradecanethioamidohexan-2-yl)carbamate (JH-T4)	Competitive inhibitor of SIRT3	Li et al. (2019a)
	3-(1H-1,2,3-triazol-4-yl) pyridine (3-TYP)	Nicotinamide competitive SIRT3 inhibitors	Galli et al. (2012)
	Selisistat (EX-527)	Nicotinamide competitive SIRT3 inhibitors	Gertz et al. (2013)
	4-(4-(Acetamidomethyl)piperidin-1-yl)thieno[3,2-*d*]pyrimidine-6-carboxamide	Promoting protein-inhibitor complex formation with SIRT3 IC50 in the range of >50 to 0.0032 μM	Disch et al. (2013),Karaman and Sippl (2015)
	4-(4-(2-Pivalamidoethyl)piperidin-1-yl)furo[3,2-*d*]pyrimidine-6-carboxamide	Promoting protein-inhbitor complex formation with SIRT3 IC50 in the range of >50 to 0.0032 μM	Karaman and Sippl (2015)
	7-(4-(2-Pivalamidoethyl)piperidin-1-yl)thieno[2,3-*c*]pyridine-2-carboxamide	Promoting protein-inhbitor complex formation with SIRT3 IC50 in the range of >50 to 0.0032 μM	Karaman and Sippl (2015)
	4-(Piperidin-1-yl)thieno[3,2-*d*]pyrimidine-6-carboxamide	Promoting protein-inhbitor complex formation with SIRT3 IC50 in the range of >50 to 0.0032 μM	Karaman and Sippl (2015)
	2-Methoxyestradiol	Binding to both the canonical and allosteric inhibitor binding sites	Gorska-Ponikowska et al. (2018)
		
SIRT4	Suramin	Suramin suppress enzyme reaction by occupying the complete active site of SIRT4	Pannek et al. (2017)
	Histone acetyltransferase inhibitor XI (SRT1720)	SRT1720 suppress SIRT4 enzymatic reaction at 100 μM of SRT1720	Pannek et al. (2017)
	Nicotinamide (NAM)	NAM suppress SIRT4 enzymatic reaction as a byproduct of sirtuin enzymatic activity	Pannek et al. (2017)
	Nicotinamide adenine dinucleotide (NADH)	NADH suppress SIRT4 enzymatic reaction by binding to co-substrate site of SIRT4	Pannek et al. (2017)
		
SIRT5	Suramin	Suramin suppress enzyme reaction by occupying the complete active site of SIRT5	Schuetz et al. (2007)
	CPS1-derived sequence	CPS-1 derived sequence serves as a SIRT5 substrate in its acetylated form	Fiorentino et al. (2022)
	Thiosuccinyllysine peptide	Thiosuccinyllysine peptide can serve as SIRT5by forming a stalled covalent intermediate during SIRT5 chemical reaction	He et al. (2012)
	Thiourea-based molecule	Hydroxyl group on the C-terminal anilide moiety of thiourea-based molecule provides an additional hydrogen bond, thereby granting tighter binding to the enzyme	Fiorentino et al. (2022)

MnSOD, manganese superoxide dismutase.

Despite understanding the conserved domains and features of mitochondrial sirtuins and unraveling their mode of action and substrate recognition, the reasons behind the differing enzymatic activities of SIRT3, SIRT4, and SIRT5 in cells remain unclear. The comprehension of NAD+ binding sites opens avenues for exploring potential modulators, providing insights into regulating and manipulating mitochondrial sirtuin activities.

## MITOCHONDRIAL SIRTUIN TARGETS AND CELLULAR FUNCTIONS

Mitochondrial sirtuins act as vital detectors, swiftly adjusting mitochondrial functions in response to fuel stress and external signals (Imai et al., 2000, Pannek et al., 2017). These sirtuins operate within feedback mechanisms, guiding specific enzymes.

### SIRT3: Major Robust Deacetylase in Mitochondrial Matrix

SIRT3 modifies proteins through acetyl post-translational changes in mitochondria, influencing mitochondrial metabolism and stress response. Truncated forms of SIRT3 protein, translocating to the mitochondria upon cellular stress, function as global mitochondrial lysine deacetylase at H4K16 in vitro and in vivo conditions. Mitochondrial sirtuins consist of SIRT3, SIRT4, and SIRT5, but only SIRT3-deficient mice exhibit striking mitochondrial protein hyperacetylation (Lombard et al., 2007). SIRT3 activates various metabolic pathways including the acyl-coenzyme A synthesis pathway, *β*-oxidation pathway, ketone body production pathway, and the electron transport chain (Hallows et al., 2006, Jing et al., 2013, Shimazu et al., 2010). Moreover, isocitrate dehydrogenase 2 involved in oxidative stress resistance is activated by increased SIRT3 expression. Besides its upregulation effect on the metabolic pathway, SIRT3 facilitates the reduction in oxidative stress and damage associated with calorie restriction via the deacetylating mitochondrial isoform of superoxide dismutase 2 (SOD2) (Qui X., et al. 2010; Choi, 2023, Qiu et al., 2010). In addition, SIRT3 also downregulates HIF-1*α* activity resulting in repressing reactive oxygen species (ROS) (Bharathi et al., 2013, Dai et al., 2014, Finley et al., 2011, Someya et al., 2010). In total, biochemical studies reveal that SIRT3 deacetylates many targets simultaneously in the mitochondrial matrix to rearrange homeostasis in response to stress conditions influencing the acetylation status of metabolic pathways.

### SIRT4: Potential Control of Mitochondrial Processes

In contrast to SIRT3 and SIRT5, SIRT4's deacylase function is less explored. SIRT4 reduces the activity of glutamate dehydrogenase (GDH) in mouse pancreatic β cells through NAD-dependent ADP-ribosylation (Haigis et al., 2006; Lüscher et al., 2018). Currently, there is some uncertainty regarding the activity of SIRT4 in ADP-ribosylation. The speed and efficiency of ADP ribosylation are lower than bacterial ADP ribosylation and SIRT4 deacylation activity. So, current research is looking into the targets of SIRT4 in modifying lysine proteins. SIRT4 affects enzymes in pyruvate catabolism, the urea cycle, and adipogenesis. It also increases the breakdown of branched-chain amino acids by activating methylcrotonyl-coenzyme A carboxylase (Anderson et al., 2017, Laurent et al., 2013, Pannek et al., 2017 Du et al., 2009). Furthermore, it uses various changes in lysine, suggesting that SIRT4 has a complex role in metabolism. Further investigations are required to elucidate the specific substrates and mechanistic details of the enzymatic activities of SIRT4.

### SIRT5: Overarching Control of Energy Metabolism

SIRT5, targeting lysine residues with acyl post-translational modifications, plays a pivotal role in mitochondrial energy metabolism. It regulates enzymes in pyruvate catabolism, the urea cycle, and ketone body synthesis. SIRT5's desuccinylation and deacylation activities affect the catalytic activities of enzymes like pyruvate dehydrogenase E1 subunit alpha 1 (PDHA), ornithine transcarbamylase (OTC), carbamoyl-phosphate synthase 1 (CPS1), argininosuccinate synthase 1 (ASSY), argininosuccinate lyase (ASL), arginase 1 (ARG1), and 3-hydroxy-3-methylglutaryl-CoA synthase 2 (HMGCS2) (Nakagawa et al., 2009; Nishida et al., 2015; Park et al., 2013; Rardin et al., 2013; Tan et al., 2014). Its diverse roles span both the mitochondrial matrix and the cytoplasm, highlighting its broad impact on cellular metabolism.

### Revealing Cellular Functions: Paths for Future Exploration

SIRT3, SIRT4, and SIRT5 showcase varied roles, fine-tuning substrates across multiple metabolic pathways. Yet, the full characterization of their substrates remains incomplete. Understanding the cellular functions driven by SIRT-dependent enzymatic activities and metabolic functions will be vital for unraveling the intricate roles of these mitochondrial sirtuins. The complexity of their actions suggests promising paths for future research, providing insights into potential therapeutic targets for metabolic disorders and related diseases.

## THE DYNAMIC ROLES OF MITOCHONDRIAL SIRTUINS IN VARIED HUMAN CANCER

**Fig. 2:**
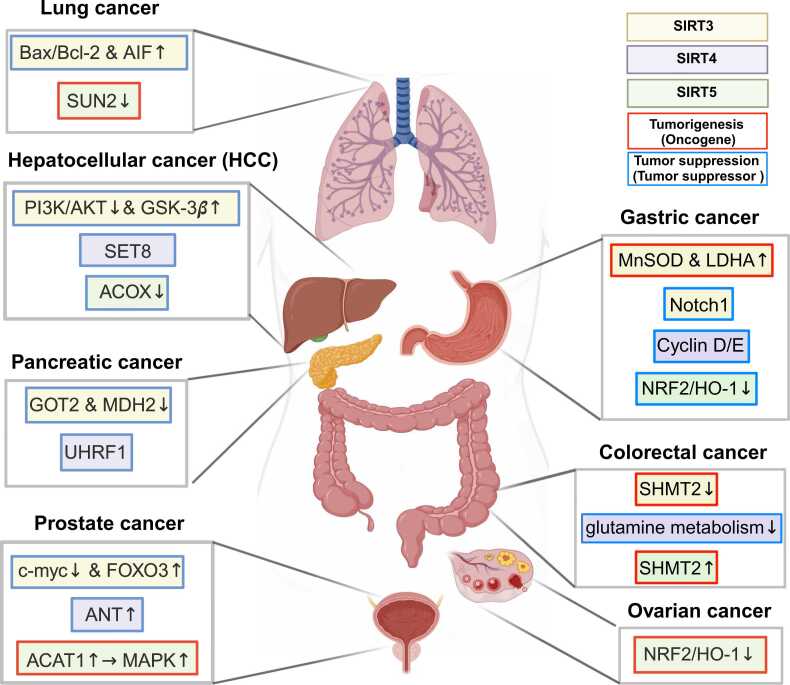
Tumorigenic and tumor suppressor roles of mitochondrial sirtuins across diverse cancer types. The schematic illustrates the potential role of SIRT3 (yellow box), SIRT4 (purple box), and SIRT5 (green box) in various human cancer types. These sirtuins function as either tumorigenic (red edge) or tumor suppressor (blue edge) across a range of human cancers, including prostate cancer, lung cancer, hepatocellular carcinoma, colorectal cancer, gastric cancer, pancreatic cancer, and ovarian cancer. AIF, apoptosis-inducing factor; SUN2, Sad1 and UNC84 domain containing 2; PI3K/AKT, phosphatidylinositol-3-kinase/protein kinase B (Akt); GSK-3*β*, glycogen synthase kinase 3 beta; SET8, methyltransferase activity (SET) domain-containing protein 8; ACOX, acyl-CoA oxidase; GOT, glutamic-oxaloacetic transaminase; MDH2, malate dehydrogenase 2; UHRF1, ubiquitin-like with plant homeodomain and ring finger domains 1; FOXO3A, Forkhead Box O3A; PAK6, p21-activated kinase 6; ANT1, adenine nucleotide translocase 1; ACAT1, acetyl-CoA acetyltransferase 1; MAPK, mitogen-activated protein kinase; MnSOD, manganese superoxide dismutase; LDHA, lactate dehydrogenase A; NRF2, nuclear factor erythroid 2-related factor 2; HO-1, heme oxygenase 1; SHMT, serine hydroxymethyltransferase.

Recent findings highlight widespread irregularities in mitochondrial sirtuins across various cancer types, indicating their involvement in crucial mechanisms like cancer metabolism, genome stability, and the tumor microenvironment. The functions of mitochondrial sirtuins in tumor development appear as either tumor suppressors or oncogenes, depending on genetic factors and the specific tumor environment (Mei et al., 2016) (Fig. 2).

In prostate cancer, Forkhead Box O3A inactivation is a hallmark linked to cancer progression (Liu et al., 2018). Dysregulation of myc proto oncogene (MYC), frequently amplified in cancers, is intricately connected to prostate cancer pathogenesis (Schaub et al., 2018). SIRT3, downregulated in prostate cancer, acts as a tumor suppressor, modulating protein kinase B and Wnt/*β*-catenin pathways, inhibiting migration and proliferation (Li et al., 2018).

In lung cancer, apoptosis evasion is mediated by upregulating BCL-2 (Lopez et al., 2022). SIRT3, a key regulator, enhances apoptosis through BCL2 Associated X, Apoptosis Regulator (Bax)/BCL2 Apoptosis Regulator (Bcl-2) ratio modulation and ROS levels (Xiao et al., 2013). SIRT5 negatively regulates Sad1 and UNC84 domain containing 2, impacting lung cancer cell dynamics (Xiao et al., 2013).

Hepatocellular carcinoma implicates the phosphatidylinositol-3-kinase/protein kinase B pathway as a survival signal (Datta et al., 1997). SIRT3 targets this pathway, restraining cancer cell proliferation and migration, and activating pro-apoptotic pathways (Song et al., 2016, Zeng et al., 2017b). SIRT4 and SIRT5 exhibit tumor-suppressive roles in hepatocellular carcinoma, impacting SET domain-containing protein 8 and acyl-CoA oxidase 1, respectively (Chen et al., 2019, Chen et al., 2018).

Colorectal cancer (CRC) reveals complex adaptation to changing environments, with serine hydroxymethyltransferase 2-mediated mitochondrial serine metabolism contributing to 5-Fluorouracil (5-FU) resistance (Pranzini et al., 2022). SIRT3, a major deacetylase in mitochondria, promotes CRC progression by deacetylating serine hydroxymethyltransferase 2 (Wei et al., 2018). In contrast, SIRT4 and SIRT5 exert opposing effects, influencing CRC metabolism and oxidative damage (Miyo et al., 2015, Yang et al., 2018).

Gastric cancer highlights the dual role of SIRT3, acting as a suppressor and promoter by modulating Notch signaling and enhancing ATP production (Cui et al., 2015, Wang et al., 2015). Additionally, SIRT4 and SIRT5 exert regulatory control over cell cycle progression and ATP production in gastric cancer cells (Hu et al., 2019; Lu et al., 2019).

Pancreatic cancer unveils SIRT3 as a tumor suppressor activating the malate-aspartate shuttle, facilitating increased ATP production, and supporting cancer cell proliferation (Yang et al., 2015). Upstream regulation of SIRT4 by ubiquitin-like with PHD and ring finger domains 1 sheds light on epigenetic mechanisms in pancreatic cancer oncogenesis (Hu et al., 2019, Hu et al., 2019).

In ovarian cancer, SIRT5 emerges as a critical player, with its increased expression correlating with poor chemotherapy response. SIRT5 contributes to cisplatin resistance by suppressing DNA damage in a ROS-dependent manner (Sun et al., 2019).

Acute myeloid leukemia (AML) cells possess metabolism profiles, such as higher rates of oxidative phosphorylation and dependence on fatty acid oxidation for survival, and are dependent on the sophisticated regulation of ROS generation for survival (O’Brien et al., 2023). One example is the sensitivity of primary AML cells to cytarabine correlated with SOD2 acetylation. The SOD2 deacetylase, SIRT3, protected AML cells from chemotherapy by inhibited apoptosis via inhibited drug-induced production of mitochondrial ROS (Ma et al., 2019). SIRT5, as a lysine deacylase, activates glutaminase which converts glutamine to glutamate metabolized to α-ketoglutarate by glutamate dehydrogenase 1 (GLUD1) or aminotransaminases (glutamic-oxaloacetic transaminase 1/2 (GOT1/2), glutamic-pyruvic transaminase 2 (GPT2), and phosphoserine aminotransferase 1 (PSAT1)) (Yan et al., 2021).

In conclusion, this study elucidates the multifaceted roles of mitochondrial sirtuins across diverse human cancers, providing insights into potential therapeutic targets and avenues for research.

## CONCLUSIONS AND FUTURE PERSPECTIVES

In this review, we discuss recent findings on mitochondrial sirtuins, focusing on their control, preferences, presence, and impact on diverse cancers. While our focus centers on SIRT3, SIRT4, and SIRT5 and their metabolic roles, fueled by the concentration of mitochondrial NAD+ in these regions, critical questions persist. Notably, uncertainties surround the distinct binding partners of mitochondrial sirtuins and the impact of NAD+ precursors on the mitochondrial NAD+ pool. The discovery of mitochondrial NAD+ transporters has opened new avenues for understanding sirtuin communication via the NAD+ pool (Luongo et al., 2020). Additionally, the metabolic byproducts of NAD+, including NAM, NADH, and Nicotinamide mononucleotide (NMN), pose questions about compartmentalization and potential transporters facilitating their entry into the mitochondrial matrix. Addressing these gaps may uncover mechanisms explaining the diverse enzymatic activities of SIRT3, SIRT4, and SIRT5, utilizing the same NAD+ cofactor. Future studies are poised to enrich our understanding of these processes and their biological significance.

Although proteomics has been extensively utilized to elucidate mitochondrial sirtuin protein interactions, it is crucial to acknowledge the inherent limitations of this approach in capturing important small molecules and achieving a comprehensive understanding of cellular processes. Metabolomics offers a snapshot of cell activities, revealing the role of small molecules in biochemical reactions. Metabolomic research on NAD+ and its precursors for mitochondrial sirtuins needs more focus. Investigating other related metabolites through metabolomic analysis will be important for understanding Sirtuin biology.

Taken together, combining both proteomics and metabolomics is crucial for understanding mitochondrial sirtuins in cancer cells. This integrated approach helps bridge the gap between protein interactions and overall cellular metabolism, providing a more complete picture of how mitochondrial sirtuins regulate energy dynamics.

## Author Contributions

H.Y. conceived the project. H.Y. and H.L. wrote the manuscript.

## Declaration of Competing Interests

The authors declare that they have no known competing financial interests or personal relationships that could have appeared to influence the work reported in this paper.
